# Sterilization Effect on Axes and Distances Between Three Implants in Curved Lines When Using Two Types of Two-Piece Intra-Oral Scan Bodies: An In Vitro Study

**DOI:** 10.3390/dj14080491

**Published:** 2026-08-06

**Authors:** Ofir Rosner, Adar Polonsky Katz, Joseph Nissan, Ela Shir Aghov, Diva Lugassy, Yifat Manor, Gil Ben-Izhack

**Affiliations:** 1 Department of Oral Rehabilitation, The Maurice and Gabriela Goldschleger School of Dental Medicine, Gray Faculty of Medical & Health Sciences, Tel Aviv University, Tel Aviv 6997801, Israel; rosnerop@yahoo.com (O.R.); adarmatatov@mail.tau.ac.il (A.P.K.); nissandr@tauex.tau.ac.il (J.N.); elashiraghov@mail.tau.ac.il (E.S.A.); 2 Department of Orthodontics, The Maurice and Gabriela Goldschleger School of Dental Medicine, Gray Faculty of Medical & Health Sciences, Tel Aviv University, Tel Aviv 699780, Israel; divaluga@mail.tau.ac.il; 3 Dental Division, Shamir (Assaf Harofeh) Medical Center, Beer Yaacov 70300, Israel; yifatmanor@gmail.com

**Keywords:** sterilization, PEEK, titanium, CAD-CAM, scan abutment, digital dentistry, intra-oral scan body, autoclave, Primescan, oral rehabilitation, dental implants

## Abstract

**Background:** The aim of this in vitro study was to examine the effect of the sterilization process on two different two-piece intra-oral scan bodies (ISBs) using three-dimensional (3D) analysis. **Methods:** Two identical 3D-printed models with three analogs in positions #12, #13, and 14# were used: two-piece titanium (Ti-Ti) and two-piece poly-ether-ether-ketone (PEEK) and Ti (PEEK-Ti) ISBs. Each model was scanned once before sterilization. A laboratory scanner (LBS) produced a reference stereolithography (STL) file, followed by 30 scans with an intra-oral scanner (IOS), producing 30 STL files. Both ISBs underwent three cycles of sterilization followed by thirty scans using an IOS, producing another 30 STL files. Each STL file was superimposed on the LBS reference STL file using 3D analyzing software, and the following parameters were calculated: inter-implant distance, intra-implant distance, inter-implant angle, and intra-implant angle. The Kolmogorov–Smirnov test indicated non-normal distribution; therefore, Wilcoxon signed-rank tests and Mann–Whitney tests were performed (*p* < 0.05). **Results:** Within groups, after sterilization, the mean errors of the Ti-Ti ISB were significantly increased regarding inter-implant distance 12_13 (*p* < 0.05), intra-implant distance 14 (*p* = 0.0005), inter-implant angle 12_13 (*p* = 0.0005), inter-implant angle 13_14 (*p* = 0.001), intra-implant angle 13 (*p* = 0.005), and intra-implant angle 14 (*p* < 0.05). Within groups, after sterilization, the mean errors of the PEEK-Ti ISB were significantly increased regarding inter-implant distance 12_13, 13_14, and 12_14 (*p* = 0.0005), intra-implant distance 12 (*p* < 0.05), intra-implant distance 13 and 14 (*p* = 0.0005), inter-implant angle 12_13 (*p* = 0.0005), and inter-implant angle 13_14 (*p* = 0.005). Between groups, after sterilization, the mean errors for the PEEK-Ti ISB were significantly higher (*p* < 0.0005) regarding all parameters compared to the Ti-Ti ISB. **Conclusions:** Both PEEK-Ti and Ti-Ti ISBs showed significant changes in most parameters after three cycles of sterilization, with the PEEK-Ti ISB exhibiting greater changes compared to the Ti-Ti ISB. Therefore, three cycles of sterilization affect axes and distances for both PEEK-Ti and Ti-Ti ISBs.

## 1. Introduction

Passive fit is one of the most important factors regarding implant-supported restorations, as it is essential for minimizing mechanical and biological complications [1,2,3]. Capturing and transferring implant positions from the mouth to a digital dental model is crucial for achieving a satisfactory passive fit of the restoration [4,5].

Intra-oral scanners (IOSs) can be used to capture implant position when using intra-oral scan bodies (ISBs), achieving a computer-aided design (CAD) mesh [6,7].

The geometry of ISBs is different between different commercial companies, but all have three main areas: a base, a body, and a scan area. Most ISBs are two-piece structures that can be made from titanium (Ti), poly-ether-ether-ketone (PEEK), or a combination of these materials [6,8]. ISBs can be reused only after following the sterilization protocol suggested by their manufacturer, which may involve steam autoclave (hydrothermal treatment) treatment under high pressure and high temperature to achieve the best sterilization results [9].

Recent studies conducted on PEEK and Ti demonstrated that sterilization processes cause three-dimensional (3D) changes that affect their mechanical properties, hence reducing the quality of the digital model [10,11,12]. In everyday dentistry, ISBs are reused and undergo different sterilization protocols. It has already been reported that the sterilization process of these materials affects their 3D structure, which leads to a reduction in the accuracy of the digital model, hence reducing passive fit [13,14,15].

Studies regarding the impact of sterilization on ISBs and the effect on accuracy of implant positions in the digital model are sparse, as this field has not been extensively investigated. Changes in the 3D position of the implant in the digital model may affect passive fit, as well as occlusal contacts and proximal contacts, leading to poor clinical results. Comparisons between two different materials (PEEK and Ti) are essential for industries to receive the most appropriate recommendation as to which material is better for reuse [16].

Superimposing digital stereolithography (STL) files is a well-known method for comparing deviations in distances and angles between implants. The laboratory scanner (LBS) is defined as the gold standard, and the STL files produced by the IOS can be superimposed on the LBS file to measure deviations [17,18].

The purpose of this in vitro study was to evaluate the effect of sterilization via autoclave on two different two-piece ISBs, Zirkonzhan (ZZ) Ti-Ti and AlphaBio (AB) PEEK-Ti, regarding changes in several parameters (intra-implant distance, intra-implant axis, inter-implant distance, and inter-implant axis), when using three implants in a curved line (positions #12, #13, and #14). This is achieved by comparing the LBS reference file with the IOS files before and after sterilization.

The null hypotheses were that no differences would be found for each ISB (within groups) and no differences would be found between the two ISBs (between groups) before and after the sterilization process, regarding the four examined parameters (inter-implant distance, intra-implant distance, inter-implant angle, and intra-implant angle).

## 2. Materials and Methods

**Figure 1:**
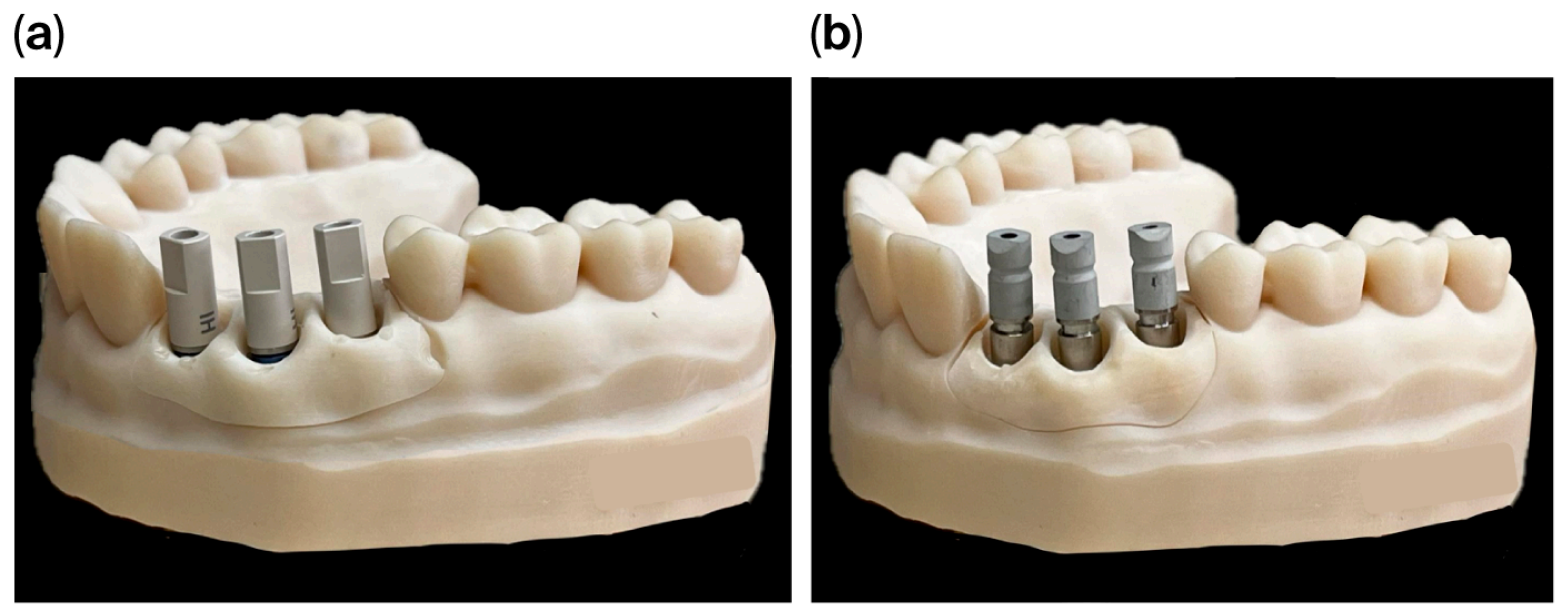
(**a**) Three PEEK-Ti (AB) ISBs in place of teeth 12, 13, 14. (**b**) Three Ti-Ti (ZZ) ISBs in place of teeth 12, 13, 14.

Two identical resin V-printed models were created using the SolFlex 650 × 350 3D printer (VOCO GmbH, Cuxhaven, Germany). In the first model, three MIS (M.I.S implants technology, Bar Lev Industrial Park, Misgav, Israel) standard internal hexagon implant digital analogs, with a diameter of 3.75 mm and a length of 11.5 mm, were used for the locations of teeth #12, 13# and #14. Three two-piece Ti-Ti (Zirkonzhan, Gais, South Tyrol, Italy) ISBs were used, all with a cylindrical asymmetric geometry and internal hexagonal connection. In the second model, three AB (Alpha-Bio Tec., Petah Tikva, Israel) standard internal hexagon implant digital analogs, with a diameter of 3.2 mm and a length of 10 mm, were used for the location of teeth #12, 13# and #14. Three two-piece PEEK-Ti (Alpha-Bio Tec., Petah Tikva, Israel) ISBs were used, all of which had cylindrical asymmetric geometry (Figure 1).

ZZ ISBs were screwed to the implant analogs using an analog torque ratchet wrench SIAK1001 (Zirkonzhan USA Inc., Norcross, GA, USA) at 15 N·cm, and AB ISBs were screwed to the implant analogs using the analog torque ratchet wrench Universal 10–45 N·cm (Alpha-Bio Tec., Petah Tikva, Israel) at 15 N·cm [19].

Both models, with ISBs screwed to the analogs, were scanned once using a LBS, the 7 Series dental wings (Dental wings^®^, Montreal, QC, Canada), and a QR file was obtained and converted to a STL file. This was referred to as the LBS reference file.

Afterwards, for each model, 30 scans were conducted, according to the manufacturer’s recommendations, by a single experienced user (G.B-I.) using Primescan (CEREC^®^ Primescan; Dentsply Sirona, Milford, DE, USA), and 30 STL files were produced for each model. Using digital software (PolyWorks^®^ 2020; InnovMetric, Québec, QC, Canada), a best-fit method was applied, and superimposition of each IOS STL file with the LBS reference file was conducted (for a total of 30 times for each model).

The ISBs were inserted into an autoclave (IOVU-752S, Tuttnauer, Bet Shemesh, Israel); the sterilization method was pre-vacuum; and three successive cycles of sterilization were applied for each of the ISBs. Both AB and ZZ had exposure times of 3 min at a temperature of 134 °C. A 20 min dry cycle was performed, as recommended by the manufacturer (steam sterilizer ISO 17665) [20].

The same methodology described above was conducted after the sterilization process. The ISBs were repositioned at a torque of 15 N·cm, and 30 scans were performed using Primescan for each model, followed by superimposition of each IOS STL file with the LBS reference file using the best-fit method.

**Figure 2:**
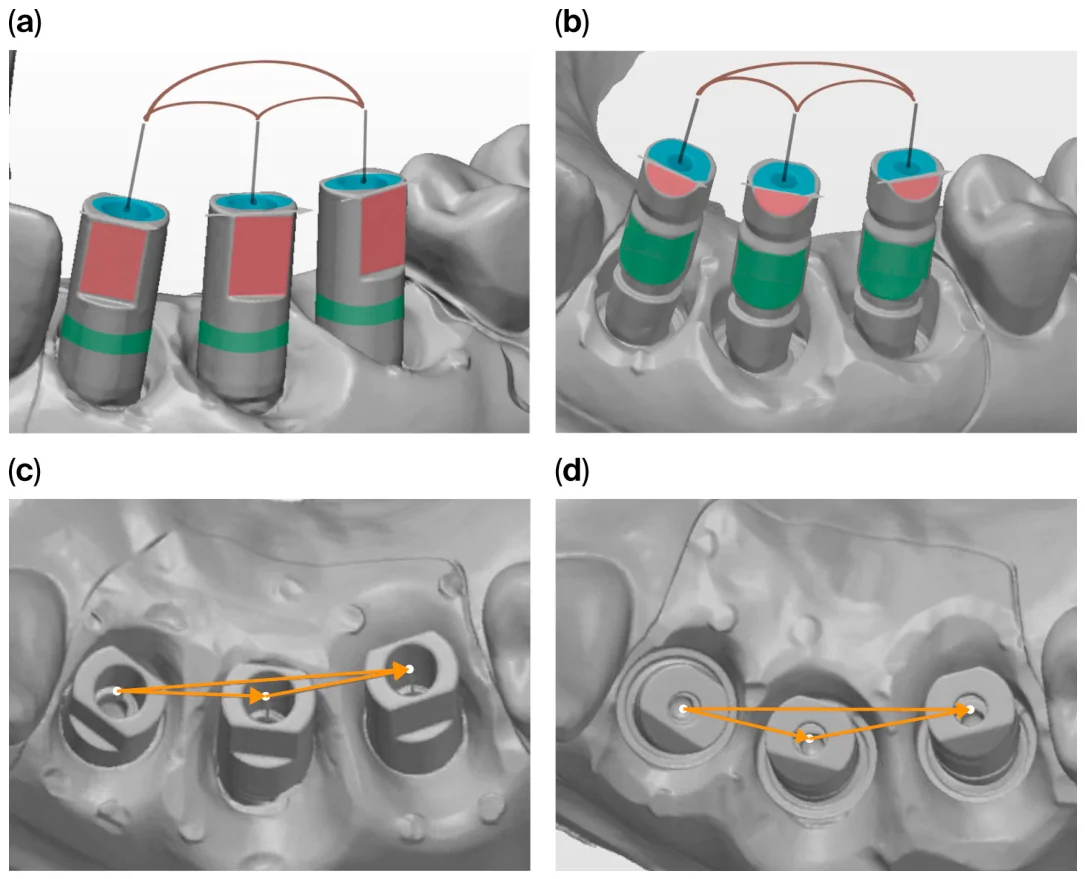
(**a**) PEEK-Ti (AB) upper plane, cylinder, axis, central point, side plane, and sideline. (**b**) Ti-Ti (ZZ) upper plane, cylinder, axis, central point, side plane, and sideline. (**c**) PEEK-Ti (AB) inter-implant distance. (**d**) Ti-Ti (ZZ) inter-implant distance.

**Figure 3:**
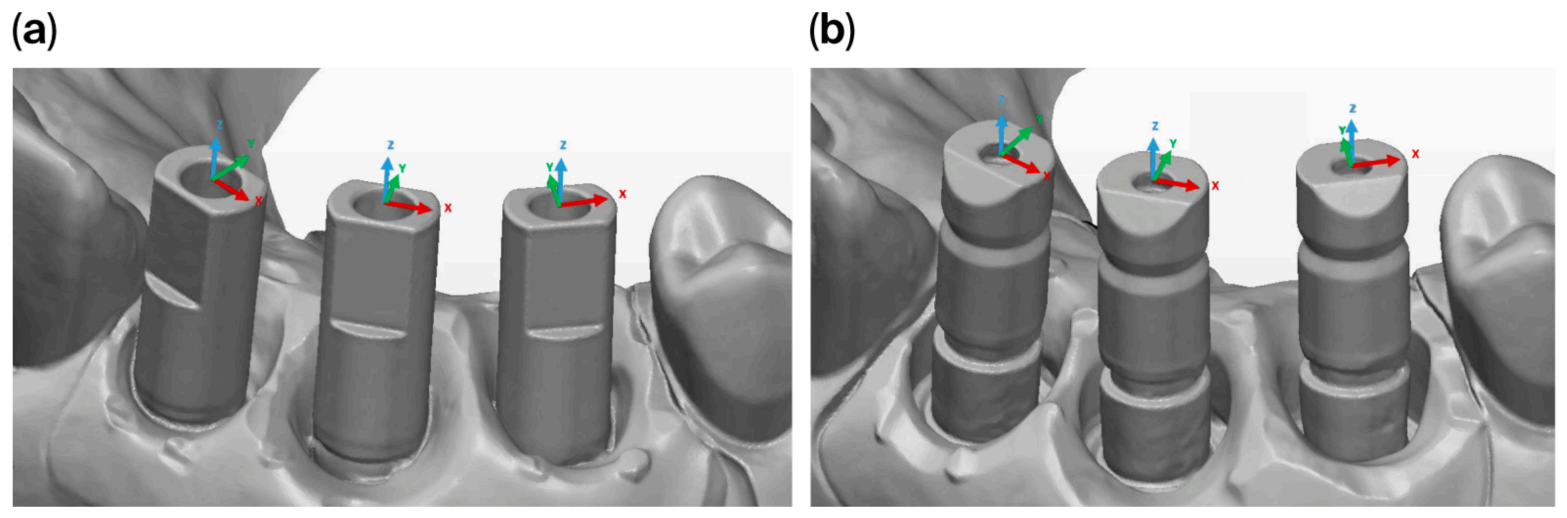
(**a**) PEEK-Ti (AB) system of axes X, Y, and Z. (**b**) Ti-Ti (ZZ) system of axes X, Y, and Z.

Both the ZZ and AB ISBs have a flat-surface geometry that points to the buccal plane (Figure 1). The superimposition of each IOS STL file with the LBS reference file was based on the adjacent teeth and on the unique geometry of both ISBs. This is an advantage as it is easier for overlapping, allowing the best-fit method to be used with all the data measured. To measure and analyze all the data, many definitions were used, allowing calculations of the differences in distances and axes between the ISBs and inside each ISB by comparing the LBS reference file and each IOS STL file. The definitions are as follows (Figure 2):

- **Upper plane** (azure plane): Occlusal surface used to achieve the best fit between the two STL files for ZZ and AB ISBs.
- **Cylinder** (greenish): Outer cylinder used to achieve the best fit between the two STL files for ZZ and AB ISBs.
- **Axis** (gray line): Longitudinal axis used to achieve the best fit between the two STL files for ZZ and AB ISBs.
- **Central point** (white dot): Point of intersection between cylinder and upper plane for both ZZ and AB ISBs.
- **Side plane** (pink plane): Side plane used to achieve the best fit between the two STL files for ZZ and AB ISBs.
- **Sideline** (purple line): Line of intersection between the upper plane and side plane, used for defining the system of axes of each ISB.
- **Inter-implant distance** (orange arrow): Distance between the central points of each ISB for both ZZ and AB. The inter-implant distances are 12_13, 13_14, and 12_14.
- **Inter-implant angle** (brown curved line): Angle between the axes of each two ISBs for both ZZ and AB. The inter-implant angles are 12_13, 13_14, and 12_14.

i. **System of Axes**: For each ZZ and AB ISB, a system of axes was defined, originating in the central point. The X-axis (red) represents the buccal–lingual plane, with a positive direction towards the buccal plane. The Y-axis (green) represents the mesial–distal plane, with a positive direction towards the distal plane. The *Z*-axis (blue) represents the occlusal–gingival plane, with a positive direction towards the occlusal plane (Figure 3).

To calculate deviations and differences, the following parameters were used from the LBS reference model file: intra-implant distance; intra-implant angle; inter-implant distance; and inter-implant angle. The two methods used were as follows:

The first method involved the superimposition of each 30 IOS STL files (for each ISB) with the LBS reference model file (for each ISB) using the PolyWorks|Inspector™ Software Verification and Measurement (PolyWorks^®^ 2020; InnovMetric, Québec, QC, Canada). The best-fit algorithm was used for the following parameters:

1. Intra-implant distance _12,13,14_ (mm)—The calculation was performed by measuring the distance between the central point of the LBS reference model file (X, Y, Z) and the counterpart central point of each IOS STL file (x, y, z). The calculation is given as follows: intra-implant distance = $\sqrt{({X-x)}^{2}+({Y-y)}^{2}+{\left(Z-z\right)}^{2}}$, (X, Y, Z = 0).
2. Intra-implant angle _12,13,14_ (angle)—The calculation was performed by measuring the 3D angle formed between the longitudinal axis of the LBS reference model file and the counterpart longitudinal axis of each IOS STL file.

The second method involved subtracting measurements between the two STL files (IOS and LBS) for the following parameters:

3. Inter-implant distance _12_13,13_14,12_14_ (mm)—Each distance was calculated by subtracting the results of each IOS STL file and LBS reference model file; superimposition was not needed for this calculation.
4. Inter-implant angle _12_13,13_14,12_14_ (angle)—Each angle was calculated by subtracting the results of each IOS STL file and LBS reference model file; superimposition was not needed for this calculation.

G* Power (3.1.9.4) analysis was conducted. To compare ZZ and AB, Mann–Whitney tests with 30 scans had high sensitivity to detect effect sizes of Cohen’s f = 0.73 with 80% power (α = 0.05). For within-group comparisons, Wilcoxon signed-rank test had high sensitivity to detect effect sizes of Cohen’s f = 0.54 with 80% power (α = 0.05).

Statistical analysis was performed using the Statistical Package for Social Sciences for Windows Release 23.0 (SPSS Inc., Chicago, IL, USA). The Kolmogorov–Smirnov test was used on study variables indicating a non-normal distribution (*p* < 0.05). Mann–Whitney tests were used for comparisons between groups, while Wilcoxon signed-rank tests were used for comparisons within groups. The statistical significance level for this work was *p* < 0.05.

## 3. Results

When the Kolmogorov–Smirnov test indicated non-normal distribution (*p* < 0.05), Mann–Whitney tests were used to evaluate differences between the two groups before and after sterilization, and Wilcoxon signed-rank tests were used to evaluate the differences within each group.

Table 1 and Table 2 show the mean errors, standard deviations (SDs), and ranges and percentiles (P25, P50, P75) of inter-implant distances 12_13,13_14, 12_14, intra-implant distances 12, 13, 14, inter-implant angles 12_13, 13_14, 12_14, and intra-implant angles 12, 13, 14 for ZZ and AB ISBs, respectively, before and after sterilization.

**Figure 4:**
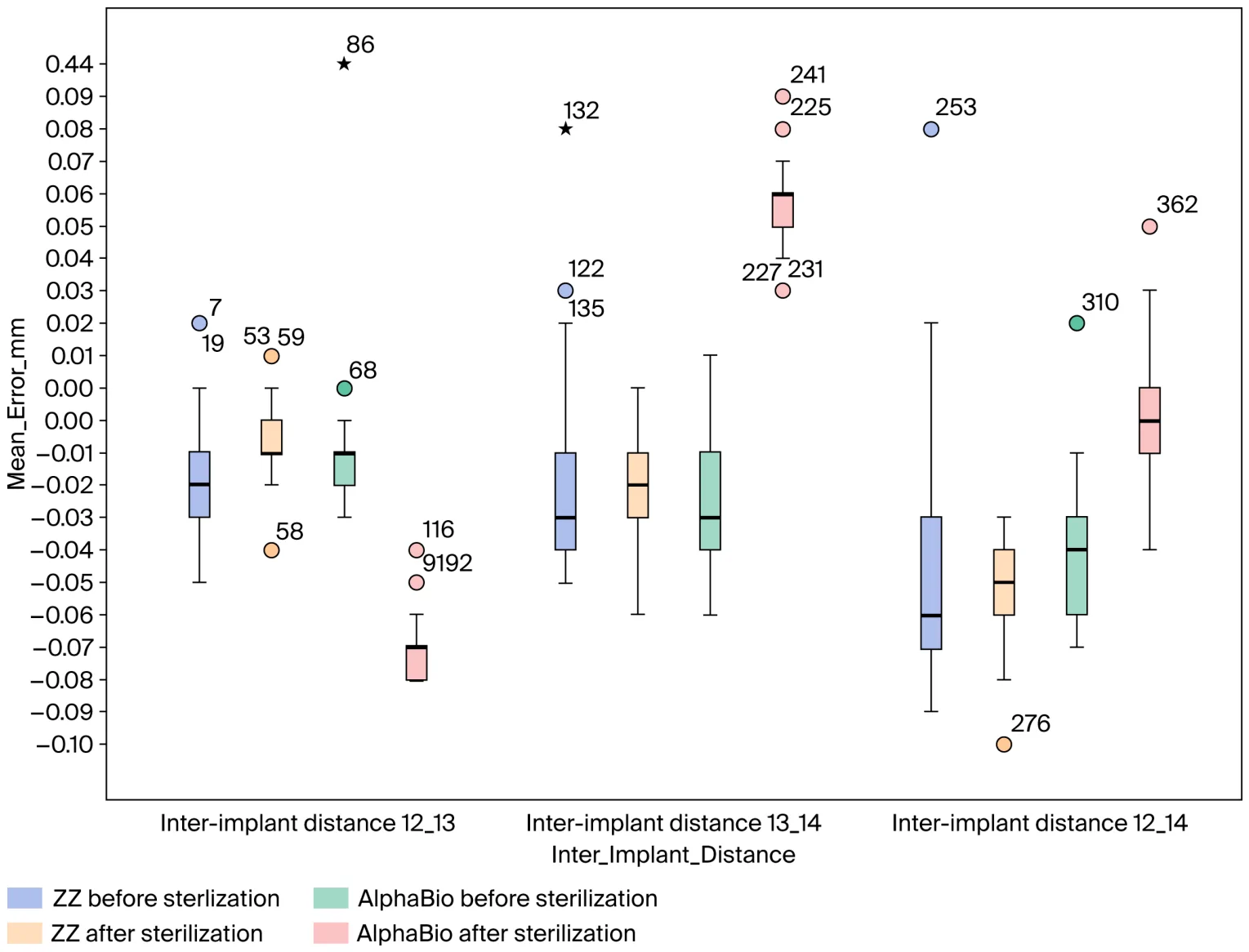
Inter-implant distance mean errors ± SDs for Ti-Ti ZZ ISB and PEEK-Ti AB ISB before and after sterilization. The symbol ‘○’ represents mild outliers (values between 1.5 and 3 times the IQR from the box), and ‘★’ represents extreme outliers (values exceeding 3 times the IQR from the box). Numbers indicated above these symbols denote the specific measurement number of each outlier.

**Figure 5:**
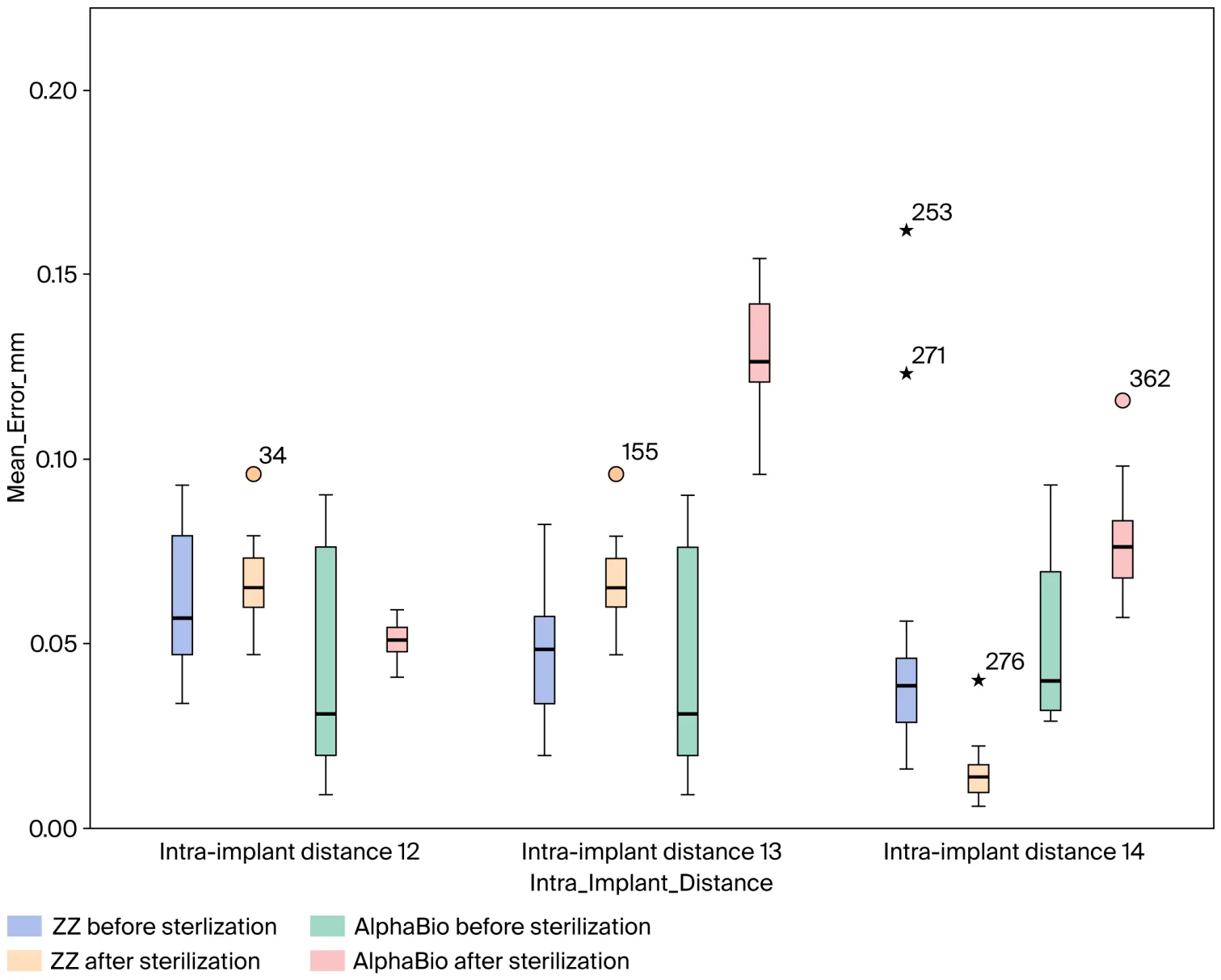
Intra-implant distance mean errors ± SDs for Ti-Ti ZZ ISB and PEEK-Ti AB ISB before and after sterilization. The symbol ‘○’ represents mild outliers (values between 1.5 and 3 times the IQR from the box), and ‘★’ represents extreme outliers (values exceeding 3 times the IQR from the box). Numbers indicated above these symbols denote the specific measurement number of each outlier.

**Figure 6:**
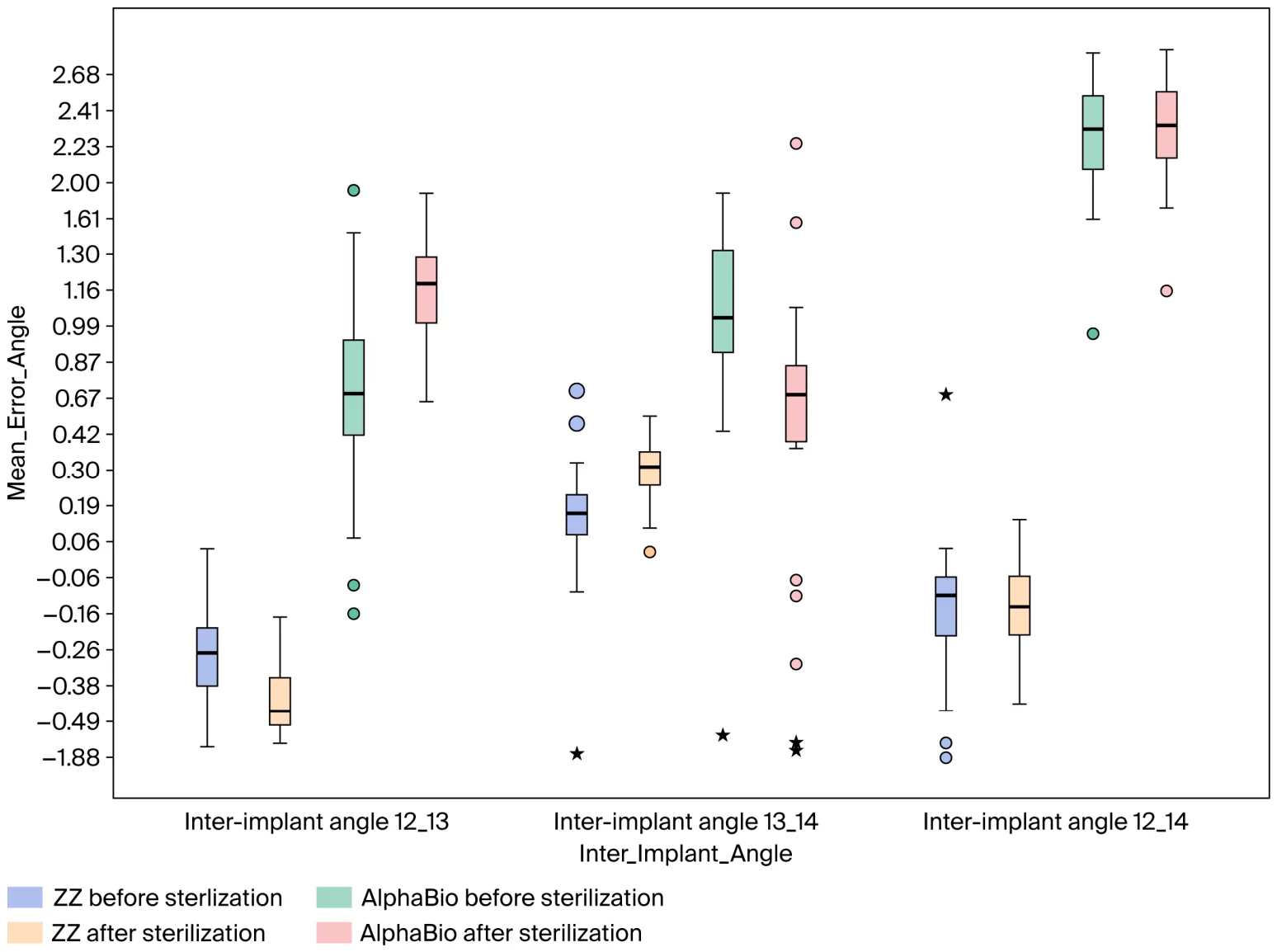
Inter-implant angle mean errors ± SDs for Ti-Ti ZZ ISB and PEEK-Ti AB ISB before and after sterilization. The symbol ‘○’ represents mild outliers (values between 1.5 and 3 times the IQR from the box), and ‘★’ represents extreme outliers (values exceeding 3 times the IQR from the box).

**Figure 7:**
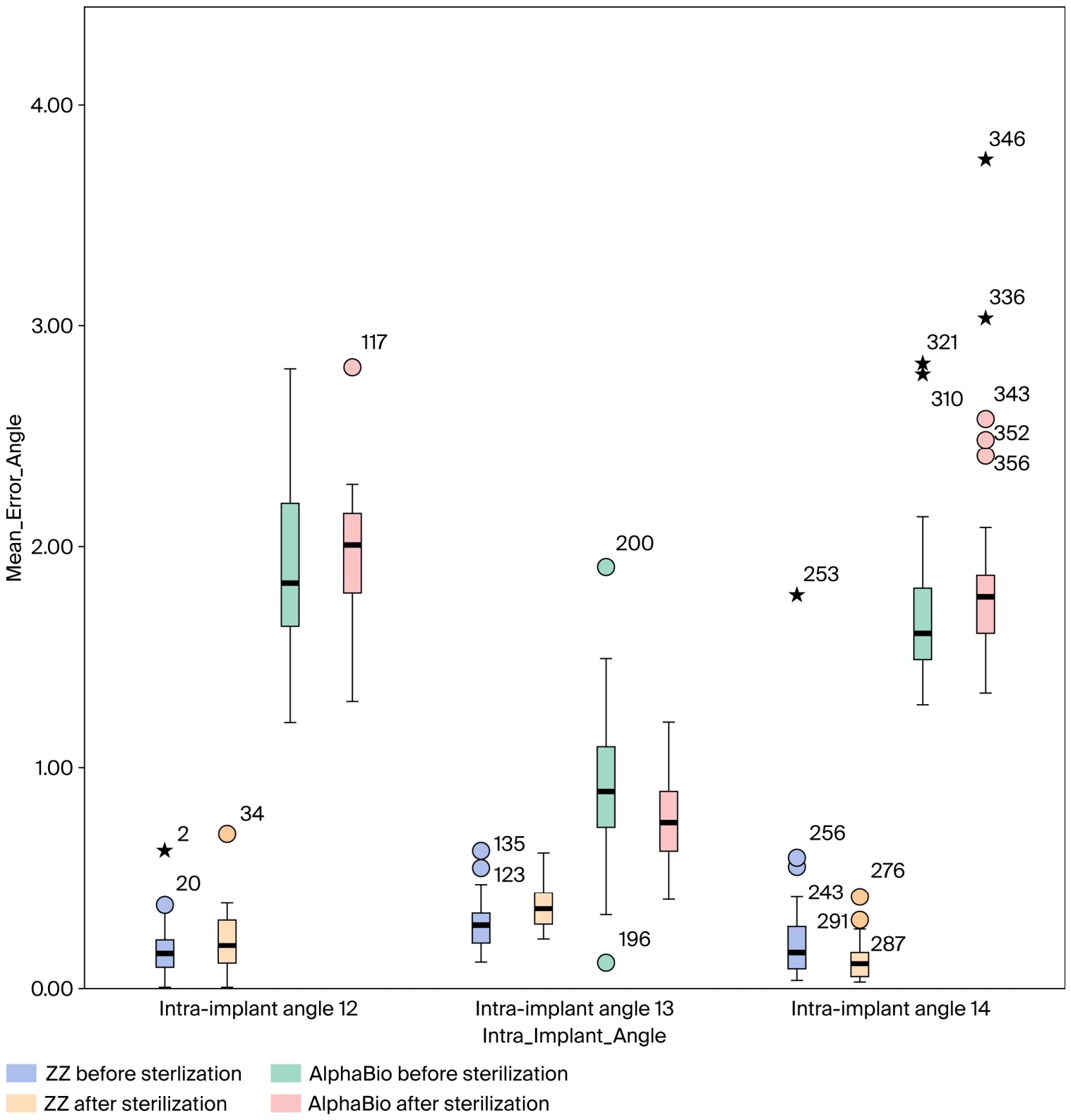
Intra-implant angle mean errors ± SDs for Ti-Ti ZZ ISB and PEEK-Ti AB ISB before and after sterilization. The symbol ‘○’ represents mild outliers (values between 1.5 and 3 times the IQR from the box), and ‘★’ represents extreme outliers (values exceeding 3 times the IQR from the box). Numbers indicated above these symbols denote the specific measurement number of each outlier.

Wilcoxon signed-rank tests showed that after sterilization of the Ti-Ti ZZ ISB, the mean errors were significantly higher for the inter-implant distance 12_13 (*p* < 0.05) (Figure 4), intra-implant distance 14 (*p* = 0.0005) (Figure 5), inter-implant angle 12_13 (*p* = 0.0005), inter-implant angle 13_14 (*p* = 0.001) (Figure 6), intra-implant angle 13 (*p* = 0.005), and intra-implant angle 14 (*p* < 0.05) (Figure 7).

No significant differences were found regarding inter-implant distance 13_14 (*p* = 0.159), inter-implant distance 12_14 (*p* = 0.651), intra-implant distance 12 (*p* = 0.388), intra-implant distance 13 (*p* = 0.497), inter-implant angle 12_14 (*p* = 0.644), and intra-implant angle 12 (*p* = 0.206).

Wilcoxon signed-rank tests showed that after sterilization of the PEEK-Ti AB ISB, the mean errors were significantly higher for the inter-implant distance 12_13 (*p* = 0.0005), inter-implant distance 13_14 (*p* = 0.0005), inter-implant distance 12_14 (*p* = 0.0005) (Figure 4), intra-implant distance 12 (*p* < 0.05), intra-implant distance 13 (*p* = 0.0005) and intra-implant distance 14 (*p* = 0.0005) (Figure 5), as well as the inter-implant angle 12_13 (*p* = 0.0005) and inter-implant angle 13_14 (*p* = 0.005) (Figure 6).

No significant differences were found regarding inter-implant angle 12_14 (*p* = 0.688), intra-implant angle 12 (*p* = 0.719), intra-implant angle 13 (*p* = 0.082), and intra-implant angle 14 (*p* = 0.071) (Figure 7).

Mann–Whitney tests were used to compare the mean errors of variables between the Ti-Ti ZZ and PEEK-Ti AB ISBs before and after sterilization.

Before sterilization, significant differences were found between the Ti-Ti ZZ and PEEK-Ti AB (PEEK-Ti AB less accurate) regarding inter-implant distance 12_13 (*p* < 0.05) (Figure 4), intra-implant distance 12 (*p* = 0.0005) (Figure 5), inter-implant angle 12_13 (*p* = 0.0005), inter-implant angle 13_14 (*p* = 0.0005), inter-implant angle 12_14 (*p* = 0.0005) (Figure 6), intra-implant angle 12 (*p* = 0.0005), intra-implant angle 13 (*p* = 0.0005) and intra-implant angle 14 (*p* = 0.0005) (Figure 7) as the mean errors were higher for the PEEK-Ti AB.

No significant differences were found regarding the inter-implant distance 13_14 (*p* = 0.162), inter-implant distance 12_14 (*p* = 0.072), intra-implant distance 13 (*p* = 0.101), and intra-implant distance 14 (*p* = 0.145).

After sterilization, significant differences were found between the Ti-Ti ZZ and PEEK-Ti AB regarding all the study variables (*p* < 0.0005 for all variables) as the mean errors were higher for the PEEK-Ti AB (Figure 4, Figure 5, Figure 6 and Figure 7).

## 4. Discussion

This in vitro study, using a previous method [15,21,22], aimed to evaluate the effect of the sterilization process on several parameters (inter-implant distance, intra-implant distance, inter-implant angle, and intra-implant angle) regarding two different two-piece ISBs (Ti-Ti ZZ and PEEK-Ti AB); the study compared IOS STL files with the LBS reference file (gold standard) before sterilization and after the sterilization process.

Both null hypotheses were rejected as significant differences were found before and after sterilization for each ISB (within groups) and significant differences were also found between the two ISBs (between groups) before and after the sterilization process (*p* < 0.05).

When examining within-group results, the sterilization process affected both ISBs, as many parameters were significantly higher after sterilization compared to before sterilization, meaning that both Ti and PEEK were affected by the sterilization process. These results are aligned with previous studies. Diker et al. showed that the sterilization process generally increased displacements of PEEK ISBs, using the same protocol as this study [23]. Other studies have also shown that PEEK increases deformations after the sterilization process [12,24]. Ben-izhack et al. showed that the sterilization process has the potential to affect the 3D structure of Ti, using the same protocol as this study [15].

When examining between-group results, the sterilization process affected PEEK-Ti ISBs more significantly for all parameters compared to Ti-Ti ISBs. These results are consistent with Arcuri et al., who reported that ISB material influences accuracy, as one-piece Ti ISBs had lower errors compared to PEEK-Ti ISBs [25].

Several studies that examined one-piece PEEK SBs and one-piece Ti SBs demonstrated different results, as Arcuri et al. [25], Völler et al. [26], and Ata et al. [20] showed favorable results for one-piece PEEK compared to one-piece Ti. By contrast, Costa Santos et al. [27] showed the opposite. It is important to notice that one-piece and two-piece SBs are different regarding their sterilization protocols, as the cement between the two pieces may be affected more than the materials during processing [28].

Another explanation for the differences between the two groups is different geometries. It is reported in the literature that geometry influences the capability of achieving better accuracy [29]. As the structure of an ISB is simpler, it confers an advantage for receiving more accurate digital scans [30]. In this study, although both ISBs had a general cylindrical geometry, the AB ISB is not completely cylindrical, and the upper surface has a rectangular shape (two flat sides). This might be a more complex shape for the IOS to scan.

Kato et al. examined the effect of sterilization on PEEK ISBs using a stone model with two implants; a sterilization process of 10 cycles was applied (135 °C for 3 min), and the same torque, 15 N·cm, was applied as in this study. Significant changes regarding the inter-implant distance and inter-implant angle were found before and after sterilization. A similar sterilization protocol was used in this study, and the results are consistent with Kato’s study, as differences were found before and after sterilization for both groups [31].

Regarding inter-implant angle, the ZZ ISB showed significantly lower mean errors compared to the AB ISB, both before and after sterilization. Almost all values of the ZZ ISB (except inter-implant angle 12_13), before and after sterilization, were within the values reported in the literature (0.07–0.3°) [32]. By contrast, all values of the AB ISB, before and after sterilization, were higher than the values reported in the literature. The significant differences between the two ISBs can be explained by the measuring technique and different geometries, as the references between the two ISBs are different [29].

When examining the inter-implant distance, before sterilization, the ZZ ISB and AB ISB differed only in regard to inter-implant distance 12_13 (with higher mean errors for AB); after sterilization, the ZZ ISB and AB ISB differed for all parameters (AB with higher mean errors). For the ZZ ISB, changes were observed from 1 to 10 μm, while for the AB ISB, changes were identified from 28 to 68 μm. In a previous study by Ben-izhack et al. [15], the same methodology was applied but for implants in a straight line (positions 15, 16, 17). Changes for the ZZ ISB were detected from 16 to 59 μm. The results obtained using a straight-line model for the ZZ ISB are more consistent with the results obtained for the AB ISB using a curved line model. This can be explained by another previous study from the same group [18], which demonstrated that when using the digital method, the results between straight- and curved-line situations are similar. Andriessen et al. [33] suggested that between two implants, the inter-implant distance deviation should be below 100 μm; in this study, all the results for inter-implant distance and deviations were below 100 μm.

The intra-implant angle is another important parameter that may affect passive fit. Andriessen et al. [32] showed that deviations over 0.194 degrees for 14.8 mm create a 50 μm difference at the apex of an implant, which is beyond the acceptable threshold; hence, a maximal deviation of 0.4 degrees is recommended. In this study, the intra-implant angles for the ZZ ISB were all below 0.4 degrees both before and after sterilization; however, for the AB ISB, all the results were above 0.4 degrees.

Sterilization may compromise the passive fit for both scan bodies (ZZ Ti-Ti and AB Ti-PEEK) by causing structural deformations, though the extent of the risk depends on material and design. Prior to sterilization, both systems achieved acceptable inter-implant distance deviations; however, post-sterilization, the titanium-based ZZ ISB maintained superior stability with minor changes, while the PEEK-Ti AB ISB exhibited significant distortions that may lead to a lack of passive fit.

Regarding the inter-implant angle, the serialization process severely compromises the potential for a passive fit when using the AB PEEK-Ti ISBs, while the ZZ Ti-Ti scan bodies demonstrated less changes. Before and after sterilization, the ZZ ISBs consistently maintain angular deviations within the strict literature threshold of 0.07° to 0.3° [32]. In contrast, the AB Ti-PEEK ISBs fail to meet the threshold what might harm passive fit standards at any point.

When using ISBs in everyday dentistry, it is very important to know what the limit of reuse is, as the literature has already proven that all types of materials undergo deformations after the sterilization process [20]. Guidelines regarding the maximum times of reuse are absent from the brochures of companies, and more research in this field is needed to understand what the upper limit of ISB reuse is.

The limitations of this in vitro study include controlled conditions without considering patient-related factors [34], the use of a single IOS and a single LBS, the inclusion of a resin rather than a metallic model [35], the use of one measuring technique [36], one 3D software [37], and only three cycles of sterilization [14], one physical model per group, 30 repeated scans rather than independent specimens [15], and the absence of direct fit evaluation and different types of implants and analogs between the two groups.

Future in vivo and in vitro studies are needed to corroborate the findings from this study when examining the maximum number of sterilizations cycles an ISB can undergo and still maintain a good clinical outcome when fabricating fixed partial dentures on implants.

## 5. Conclusions

Within the limitations of the different implant and analog systems evaluated, the following conclusions were drawn specifically for AB and ZZ scan bodies:

1. Both PEEK-Ti and Ti-Ti ISBs showed significant changes in most parameters after three sterilization cycles.
2. Within the specific commercial assemblies and implant analog systems tested, the AB PEEK-Ti ISB exhibited higher mean errors after three sterilization cycles than the ZZ Ti-Ti ISB.
3. Three cycles of sterilization affect axes and distances for both PEEK-Ti and Ti-Ti ISBs.
4. Findings are preliminary and should be confirmed by future studies with more independent specimens.
5. Clinical protocols regarding the maximum number of reuses are missing.

## Figures and Tables

**Table 1:** Mean errors ± SDs, ranges, P25 (25 percentile), P50 (50 percentile, median), and P75 (75 percentile) of inter-implant distances 12–13, 13–14, 12–14, intra-implant distances 12, 13, 14, inter-implant angles 12–13, 13–14, 12–14, and intra-implant angles 12, 13, 14 of Ti-Ti ZZ ISB before and after sterilization.

	Ti-Ti ZZ ISB Before Sterilization				Ti-Ti ZZ ISB After Sterilization			
	Mean (±SD) Range	P25	P50 (Median)	P75	Mean (±SD) Range	P25	P50 (Median)	P75
Inter-implant distance 12–13 (mm)	−0.018 (±0.017) 0.077	−0.028	−0.022	−0.005	−0.008 (±0.010) 0.047	−0.014	−0.009	−0.003
Inter-implant distance 13–14 (mm)	−0.018 (±0.027) 0.128	−0.035	−0.030	−0.011	−0.019 (±0.013) 0.060	−0.026	−0.017	−0.010
Inter-implant distance 12–14 (mm)	−0.048 (±0.035) 0.167	−0.074	−0.060	−0.029	−0.050 (±0.015) 0.073	−0.056	−0.049	−0.040
Intra-implant distance 12 (central point 1) (mm)	0.062 (±0.018) 0.059	0.047	0.057	0.080	0.066 (±0.009) 0.049	0.059	0.065	0.073
Intra-implant distance 13 (central point 2) (mm)	0.047 (±0.015) 0.062	0.033	0.048	0.057	0.049 (±0.007) 0.033	0.043	0.049	0.053
Intra-implant distance 14 (central point 3) (mm)	0.043 (±0.028) 0.146	0.028	0.038	0.046	0.014 (±0.006) 0.034	0.010	0.014	0.017
Inter-implant angle 12–13 (angle)	−0.277 (±0.151) 0.737	−0.378	−0.254	−0.192	−0.430 (±0.117) 0.486	−0.499	−0.428	−0.359
Inter-implant angle 13–14 (angle)	−0.124 (±0.328) 2.124	0.089	0.163	0.225	0.301 (±0.105) 0.480	0.242	0.305	0.361
Inter-implant angle 12–14 (angle)	−0.176 (±0.38) 2.57	−0.217	−0.112	−0.055	−0.154 (±0.144) 0.578	−0.225	−0.146	−0.062
Intra-implant angle 12 (angle)	0.185 (±0.121) 0.614	0.105	0.160	0.221	0.214 (±0.141) 0.690	0.118	0.197	0.305
Intra-implant angle 13 (angle)	0.301 (±0.114) 0.496	0.212	0.289	0.343	0.374 (±0.089) 0.377	0.297	0.364	0.430
Intra-implant angle 14 (angle)	0.257 (±0.320) 1.744	0.095	0.164	0.280	0.138 (±0.102) 0.394	0.059	0.114	0.163

**Table 2:** Mean errors ±SDs, ranges, P25 (25 percentile), P50 (50 percentile, median), and P75 (75 percentile) of inter-implant distances 12–13, 13–14, 12–14, intra-implant distances 12, 13, 14, inter-implant angles 12–13, 13–14, 12–14, and intra-implant angles 12, 13, 14 of PEEK-Ti AB ISB before and after sterilization.

	PEEK-Ti AB ISB Before Sterilization				PEEK-Ti AB ISB After Sterilization			
	Mean (±SD) Range	P25	P50 (Median)	P75	Mean (±SD) Range	P25	P50 (Median)	P75
Inter-implant distance 12–13 (mm)	0.0008 (±0.083) 0.469	−0.020	−0.013	−0.008	−0.069 (±0.010) 0.042	−0.077	−0.073	−0.065
Inter-implant distance 13–14 (mm)	−0.028 (±0.015) 0.062	−0.041	−0.032	−0.013	0.056 (±0.013) 0.061	0.048	0.054	0.063
Inter-implant distance 12–14 (mm)	−0.042 (±0.019) 0.087	−0.057	−0.045	−0.030	−0.000 (±0.015) 0.085	−0.010	−0.003	0.005
Intra-implant distance 12 (central point 1) (mm)	0.042 (±0.016) 0.059	0.031	0.038	0.057	0.051 (±0.004) 0.018	0.048	0.051	0.054
Intra-implant distance 13 (central point 2) (mm)	0.041 (±0.026) 0.081	0.020	0.031	0.076	0.128 (±0.014) 0.058	0.119	0.124	0.142
Intra-implant distance 14 (central point 3) (mm)	0.050 (±0.022) 0.064	0.032	0.040	0.071	0.077 (±0.011) 0.059	0.068	0.076	0.083
Inter-implant angle 12–13 (angle)	0.707 (±0.453) 2.072	0.411	0.758	0.940	1.158 (±0.272) 1.192	0.998	1.167	1.292
Inter-implant angle 13–14 (angle)	1.07 (±0.450) 2.47	0.911	1.144	1.314	0.592 (±0.633) 3.522	0.401	0.685	0.877
Inter-implant angle 12–14 (angle)	2.25 (±0.389) 2.15	2.105	2.301	2.465	2.343 (±0.433) 2.488	2.149	2.285	2.503
Intra-implant angle 12 (angle)	1.905 (±0.382) 1.59	1.646	1.838	2.199	1.927 (±0.334) 1.51	1.78	2.009	2.148
Intra-implant angle 13 (angle)	0.912 (±0.352) 1.79	0.722	0.893	1.101	0.784 (±0.206) 0.791	0.627	0.753	0.902
Intra-implant angle 14 (angle)	1.710 (±0.364) 1.54	1.483	1.611	1.809	1.912 (±0.510) 2.417	1.610	1.775	1.918

## Data Availability

The original contributions presented in this study are included in the article. Further inquiries can be directed to the corresponding author.

## Abbreviations

ZZ	Zirkonzhan
AB	AlphaBio
Ti	titanium
PEEK	poly-ether-ether-ketone
STL	stereolithography
3D	three-dimensional
CAD	computer-aided design
IOS	intra-oral scanner
IOSs	intra-oral scanners
LBS	laboratory scanner
ISB	intra-oral scan body
ISBs	intra-oral scan bodies
